# Generalizable cancer detection from ultra-low-pass WGS via deep contextual modeling of cfDNA sequences

**DOI:** 10.1186/s43556-026-00538-w

**Published:** 2026-08-12

**Authors:** Yang Xu, Song Wang, Guofeng Sun, Yi Shen, Shuang Chang, Peng He, Shuyu Wu, Shanshan Yang, Yuan Mao, Zheng Wang, Jiaqi Liu, Tao Wang, Bingzhong Zhang, Kuirong Jiang, Jierong Chen, Hairong Huang, Junjie Peng, Xiaomeng Xia, Yumei Wu, Ming Wang, Shun Xu, Ji Tao, Li Chong, Junfei Zhu, Daxin Huang, Yuan Jiang, Yongquan Wang, Hua Bao, Xue Wu, Yang Shao

**Affiliations:** 1 Geneseeq Research Institute, Nanjing Geneseeq Technology Inc., Nanjing, China; 2 The Fourth Affiliated Hospital of Nanjing Medical University, Nanjing, China; 3 Department of Liver Surgery and Transplantation, Liver Cancer Institute, Zhongshan Hospital, Fudan University, Shanghai, China; 4 State Key Laboratory of Molecular Oncology, National Cancer Center, Cancer Hospital of the Chinese Academy of Medical Sciences, Beijing, China; 5 Department of Thoracic Surgery, Nanjing Drum Tower Hospital, Nanjing, China; 6 Department of Gynecologic Oncology, Sun Yat-sen Memorial Hospital, Sun Yat-sen University, Guangzhou, China; 7 Pancreas Center, The First Affiliated Hospital of Nanjing Medical University, Nanjing, China; 8 Department of Clinical Laboratory, Guangdong Provincial People’s Hospital, Guangzhou, China; 9 Department of Thoracic Surgery, Eastern Theater Command Hospital, Nanjing, China; 10 Department of Colorectal Surgery, Fudan University Shanghai Cancer Center, Shanghai, China; 11 Department of Gynaecology, Second Xiangya Hospital of Central South University, Changsha, China; 12 Department of Gynecologic Oncology, Beijing Obstetrics and Gynecology Hospital, Beijing, China; 13 Department of Thoracic Surgery, The First Affiliated Hospital of China Medical University, Shenyang, China; 14 Department of Gastrointestinal Medical Oncology, Harbin Medical University Cancer Hospital, Harbin, China; 15 Department of Respiratory Medicine, First People’s Hospital of Changzhou, Third Affiliated Hospital of Soochow University, Changzhou, China; 16 Department of Respiratory and Critical Care Medicine, Taizhou Central Hospital (Taizhou University Hospital), Taizhou, 318000 China; 17 Department of Radiation Oncology, Fujian Medical University Union Hospital, Fuzhou, China; 18 Clinical Laboratory of Hunan Cancer Hospital, Affiliated Cancer Hospital of Xiangya School of Medicine, Central South University, Changsha, China; 19 Department of Urology, The First Affiliated Hospital of Army Medical University, Chongqing, China; 20 School of Public Health, Nanjing Medical University, Nanjing, China

**Keywords:** Large language model, Ultra-low-pass WGS, Cell-free DNA, Fragmentomics, Low tumor fraction

## Abstract

Ultra-low-pass whole-genome sequencing (ULP-WGS) of cell-free DNA (cfDNA) offers a cost-efficient strategy for cancer detection, but its clinical application is limited by extreme data sparsity and poor model generalization. We developed Fragmentia-AI™ WGS, a mutation-calling-independent framework that uses a transformer-based multiple-instance learning architecture with sequential fine-tuning across tumor fraction (TF) strata to extract latent cancer-associated signals from ULP-WGS data. Model performance was evaluated in multiple independent cohorts, including a pan-cancer test set covering 17 cancer types, an external public dataset generated on a different sequencing platform, and a technical variability cohort with heterogeneous pre-analytical and experimental conditions. Clinical relevance was assessed by correlating model predictions with progression-free survival (PFS) in patients with advanced non-small cell lung cancer receiving chemoimmunotherapy. Sequential fine-tuning across TF strata significantly improved performance in low-TF samples, achieving a 35.6% relative increase in AUC compared with high-TF-only training (0.884 vs. 0.652). In the independent test cohort, the model achieved an overall AUC of 0.930, with consistent performance across TF strata and cancer types. External validation confirmed robust cross-platform generalizability (AUC: 0.929; sensitivity: 0.78; specificity: 0.92). The model maintained stable classification performance despite score fluctuations associated with pre-analytical and technical variables. Importantly, model-negative status, defined as a prediction score below the training-derived cutoff, remained significantly associated with improved PFS compared with model-positive status (HR = 0.49, 95% CI: 0.29–0.82) after multivariable adjustment. Collectively, this framework enables robust cancer detection and clinically meaningful risk stratification from highly sparse cfDNA sequencing data.

**Supplementary Information:**

The online version contains supplementary material available at 10.1186/s43556-026-00538-w.

## Introduction

Cell-free DNA (cfDNA)-based liquid biopsy has emerged as a promising approach for non-invasive cancer detection and disease monitoring. Whole-genome sequencing (WGS) captures fragmentation patterns, copy number variations, and mutations [1–3], but population-scale deployment remains constrained by the cost of deep sequencing. Low-pass WGS (LP-WGS, typically 0.1X–1X coverage) offers a cost-effective alternative. Fragmentomic approaches using cfDNA fragmentation profiles and motif frequencies can recover cancer signals from shallow data without high-depth mutation calling [4, 5].

Despite these advances, a critical challenge arises when sequencing depth is further reduced to the ultra-low-pass (ULP) range at approximately 0.05–0.1X. At such ultra-shallow depths, stochastic sampling noise dominates, substantially diminishing the reliability of conventional fragmentomic profiling and somatic variant detection, as the probability of capturing any specific mutation approaches zero [3, 4, 6]. Existing methodologies therefore encounter a “sensitivity floor” that limits ultra-low-cost screening. Overcoming this barrier requires approaches that detect latent, high-dimensional cancer signals in sparse genomic sequences rather than relying on explicit feature engineering.

Transformer-based large language models (LLMs) provide a potential framework for this challenge. Genomic language models such as DNABERT learn sequence “grammar” through self-supervised pre-training [7–9], while attention mechanisms capture contextual dependencies relevant to genomic structure and function [10, 11]. These models may therefore extract cancer signals from ultra-sparse reads by analyzing sequence context rather than relying on the coverage accumulation required by conventional methods. However, the application of genomic language models to cfDNA-based cancer detection presents substantial challenges in generalizability and clinical translation. AI-based genomic classifiers can overfit cohort-specific differences in sequencing platforms, library preparation, and population backgrounds [12–14]. Moreover, discrimination metrics such as the area under the receiver operating characteristic curve (AUC), while informative for benchmarking, do not directly translate into clinically actionable decision-making. In real-world screening and diagnostic settings, model deployment requires prespecified and fixed operating thresholds to ensure stable performance, reproducibility across cohorts, and interpretability for clinical use. In addition, whether prediction scores derived from ULP cfDNA sequencing carry prognostic relevance remains largely unexplored. Establishing associations between model-derived scores and clinical outcomes is critical for demonstrating that the captured signals reflect biologically meaningful tumor burden rather than technical artifacts.

In this study, we present Fragmentia-AI™ WGS, a mutation-calling-independent, reference-genome-based fragmentomic pipeline for pan-cancer detection from ULP-WGS. Sequential fine-tuning across high-, medium-, and low-tumor fraction (TF) samples was used to improve signal extraction across tumor burdens. Model performance was evaluated at a fixed predefined cutoff in independent internal and external cohorts, including the cross-platform DELFI dataset. We further assessed robustness under diverse pre-analytical and experimental conditions and examined associations between prediction scores and outcomes in advanced NSCLC treated with first-line chemoimmunotherapy. Together, these analyses define a cost-efficient, clinically interpretable, and externally generalizable ULP-WGS framework.

## Results

### Overview of study design

**Fig. 1:**
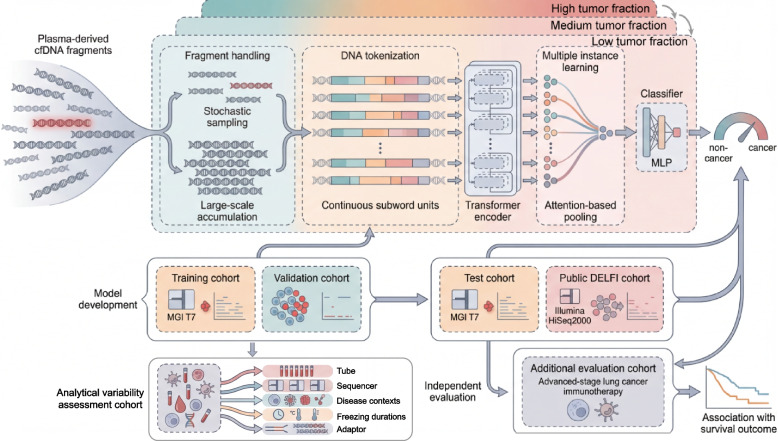
Overview of the analytical workflow. Plasma-derived cfDNA fragments were analyzed using a reference-genome-based fragmentomic pipeline. After stochastic sampling and large-scale fragment accumulation, cfDNA fragments were tokenized and encoded by a Transformer encoder to generate fragment-level embeddings. A multiple-instance learning framework with attention-based pooling aggregated fragment embeddings into a sample-level representation, which was then classified by a multilayer perceptron to predict cancer versus noncancer status. Model development was performed using the training and validation cohorts sequenced on the MGI T7 platform, followed by evaluation in an independent test cohort and an external public DELFI cohort sequenced on the Illumina HiSeq 2000 platform. Sequential fine-tuning was applied across high-, medium-, and low-tumor fraction settings to improve performance across varying levels of tumor-derived cfDNA abundance. Additional analytical variability and clinical evaluation cohorts were used to assess technical robustness and the association between model scores and clinical outcomes

This study used a reference-genome-based cfDNA strategy compatible with ULP-WGS (Fig. 1). Plasma cfDNA reads were aligned to the human reference genome, and cancer-associated signals were inferred from fragmentomics patterns related to nucleosome positioning, chromatin accessibility, and cell-of-origin differences. Aligned fragments were stochastically sampled, converted into model-ready inputs using BPE, and encoded by a Transformer. Because labels were available only at the sample level, an MIL framework treated each sample as a bag of fragment instances. Attention-based pooling aggregated fragment embeddings into a sample-level representation for MLP classification.

Model fitting used the training cohort (*n* = 3,695), and hyperparameter optimization and model selection used the internal validation cohort (*n* = 2,043; Table S1). The final model was evaluated in an independent test cohort (*n* = 5,996; Table S2) and externally validated in the public DELFI dataset (*n* = 538). We compared three TF-aware fine-tuning schemes: high-TF only, high/medium-TF, and high/medium/low-TF. TF strata were defined using ichorCNA-estimated tumor fraction as low (< 3%), medium (3–5%), or high (> 5%). The 3% cutoff was selected based on the original ichorCNA study [15], which reported 0.03 tumor fraction as the lower limit for detecting tumor presence from ULP-WGS cfDNA data, whereas 5% defined a higher-abundance stratum for ordered comparison.

Low-TF samples accounted for most cancer samples in all three cohorts, representing 66.1%, 62.3%, and 71.6%, respectively (Fig. S1a-c). Stage distribution analysis showed that low-TF samples were predominantly early-stage tumors, with stage I/II tumors comprising 77.4%, 77.4%, and 77.0% of low-TF samples in the three cohorts (Fig. S1d-f). By contrast, stage IV disease was more enriched in the high- and medium-TF groups, consistent with the expected association between higher tumor burden and increased ctDNA abundance. We also examined cancer type composition across TF strata. Low-TF samples included a broad spectrum of tumor types rather than being restricted to a single cancer type, with lung, gastric, cervical, colorectal, kidney, pancreatic, and head-and-neck cancers representing the major components across cohorts (Fig. S2). These results indicate that the low-TF subgroup reflected both early-stage enrichment and broad cancer-type representation.

To assess whether WGS-defined low-TF samples represented a biologically low-ctDNA setting, we analyzed an additional independent paired-sequencing cohort comprising samples with both ULP-WGS and matched targeted panel sequencing data (GeneseeqPrime™, 425-gene panel; *n* = 206). This cohort was not used for model training, hyperparameter optimization, threshold determination, or model selection and was used only to evaluate the biological relevance of the WGS-defined low-TF subgroup. All samples in this cohort had ichorCNA-estimated TF < 3%. The median panel-derived variant allele frequency (VAF) was 0.50% (interquartile range [IQR]: 0.36%−0.79%), indicating low but detectable tumor-derived ctDNA abundance despite low WGS-estimated TF (Fig. S3a). Model sensitivity increased with panel-derived VAF, from 72.7% in samples with VAF < 0.2% to 100.0% in samples with VAF > 5% (Fig. S3b). These findings support the biological relevance of the WGS-defined low-TF subgroup.

To evaluate model robustness, we included an analytical variability cohort covering blood collection tubes, sequencing instruments, disease contexts, plasma freezing durations, library adaptors, and technical repeats. Technical reproducibility was assessed using four aliquots from the same blood tube processed by different operators across two sequencing batches. Finally, prediction scores measured at radiographic response assessment were examined for association with progression-free survival (PFS) in 143 patients with advanced-stage NSCLC who achieved complete or partial response (CR/PR) after first-line platinum-based chemotherapy plus immunotherapy (Table S3).

### Model fine-tuning based on tumor fractions

Because lower TF reduces tumor-derived cfDNA signals, we compared three fine-tuning schemes to determine whether sequential TF-aware training improved robustness across heterogeneous samples.

When the model was trained and fine-tuned on high-TF samples alone, it achieved an AUC of 0.982 in the training cohort, which consisted exclusively of high-TF samples (Fig. S4a). In the validation cohort, performance remained high in high-TF samples (AUC: 0.988) but declined with decreasing TF (medium TF: 0.905; low TF: 0.652), yielding an overall AUC of 0.769 (Fig. S4b, c). Despite this, cancer samples consistently showed higher prediction scores than noncancer samples across cohorts and TF strata (Fig. S4d-g). Adding medium-TF samples preserved strong high-TF performance and modestly improved medium-TF discrimination, but low-TF performance remained limited (AUC: 0.702) (Fig. S5a-c). Prediction scores increased monotonically with TF (Fig. S5d, e), indicating that low TF remained challenging despite inclusion of intermediate-TF samples during fine-tuning.

**Fig. 2:**
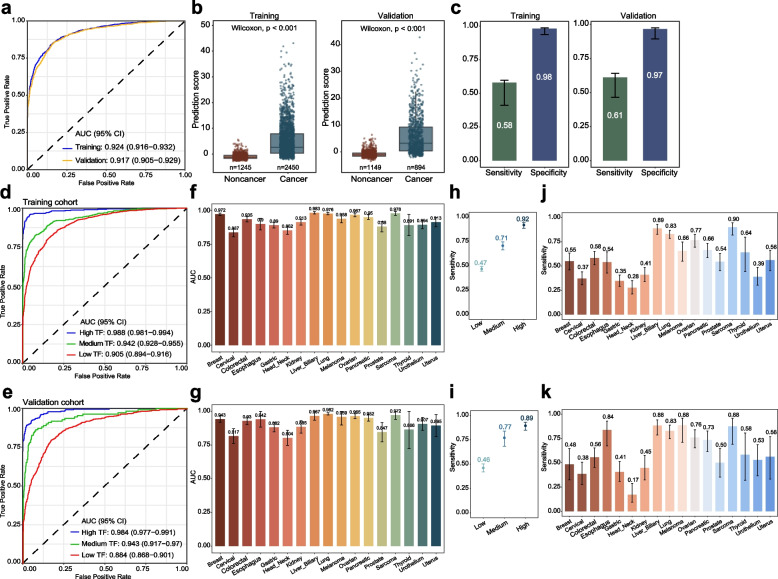
Model performance in the training and validation cohorts after fine-tuning on low-TF samples. **a** Receiver operating characteristic (ROC) curves for cancer detection in the training and validation cohorts. **b** Distribution of model prediction scores between noncancer and cancer samples in the training and validation cohorts. **c** Sensitivity and specificity at the predefined operating threshold corresponding to 98% specificity in the training cohort. **d**, **e** ROC curves stratified by tumor fraction (TF; high, medium, and low) in the training (**d**) and validation (**e**) cohorts. **f**, **g** Cancer type-specific areas under the ROC curve (AUCs) in the training (f) and validation (**g**) cohorts. **h**, **i** Sensitivity across TF strata at the predefined cutoff in the training (**h**) and validation (**i**) cohorts. **j**, **k** Cancer type-specific sensitivity at the predefined cutoff in the training (**j**) and validation (**k**) cohorts

After fine-tuning across high-, medium-, and low-TF samples, the model achieved AUCs of 0.924 (95% CI: 0.916–0.932) and 0.917 (95% CI: 0.905–0.929) in the training and validation cohorts, respectively (Fig. 2a). Prediction scores were higher in cancer than in noncancer samples in both cohorts (Wilcoxon test, *p* < 0.001; Fig. 2b). At the prespecified cutoff corresponding to 98% specificity in training (score = 1.4650357), sensitivity was 0.58 and 0.61 in the two cohorts (Fig. 2c). Performance remained robust across TF strata and cancer types, with expected degradation in low-TF settings (Fig. 2d-i; Fig. S6a-d). Low-TF AUC increased to 0.905 (95% CI: 0.894–0.916) in training and 0.884 (95% CI: 0.868–0.901) in validation, representing a marked improvement over the previous models (Fig. 2d, e). Cancer-type-specific AUCs were broadly high (Fig. 2f, g), and sensitivity increased with TF while remaining above 0.46 in low-TF samples (Fig. 2h-k).

Because early-stage low-TF cancers represent one of the most challenging scenarios for cfDNA-based detection, we evaluated sensitivity in low-TF samples stratified by clinical stage and cancer type. The model achieved sensitivities of 39.6%, 39.7%, and 39.1% in stage I/II low-TF cancers from the training, validation, and test cohorts, respectively, whereas sensitivities were higher in stage III/IV low-TF cancers, reaching 71.6%, 68.3%, and 70.8%, respectively (Table S4). Sensitivity also varied across cancer types within the low-TF subgroup (Fig. S7). Lung and liver/biliary cancers showed consistently higher sensitivity across cohorts, whereas gastric, cervical, and head-and-neck cancers showed lower sensitivity despite similarly low tumor fractions. Collectively, these analyses showed that detection performance in low-TF cancers is influenced by TF, clinical stage, and tumor type. Although the model identified a subset of early-stage low-TF cancers, these weak-signal settings remain a major challenge for cfDNA-based screening and an important focus for future improvement.

**Fig. 3:**
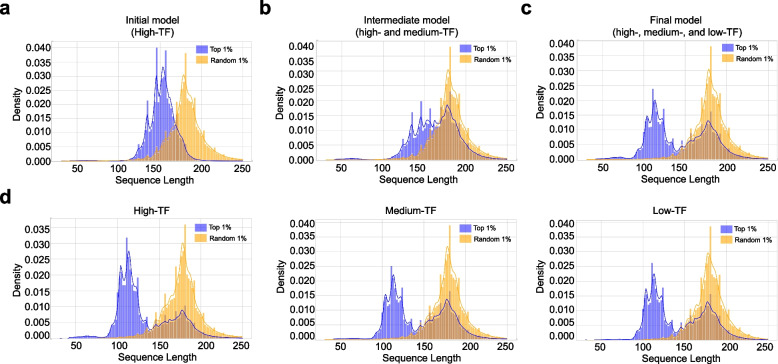
Fragment length distributions of cfDNA fragments prioritized through sequential tumor fraction fine-tuning. **a-c** Fragment length density distributions of the top 1% of cfDNA fragments prioritized by models fine-tuned using high-TF samples only (**a**), high- and medium-TF samples (**b**), and high-, medium-, and low-TF samples (**c**). For each model, the top 1% of prioritized fragments were compared with 1% randomly selected fragments from the corresponding model outputs in a randomly selected subset of 50 validation samples. **d** Fragment length density distributions of the top 1% cfDNA fragments prioritized by the final model compared with 1% randomly selected fragments in the full validation cohort, stratified by TF level

We also examined fragment length distributions of cfDNA fragments prioritized by the fine-tuning strategies in validation samples. The high-TF-only model prioritized fragments with a relatively narrow, near-unimodal length distribution, suggesting capture of dominant high-TF fragmentomic signals (Fig. 3a). Incorporating medium-TF samples broadened the distribution and increased shorter-fragment contributions, without a clear short-fragment peak (Fig. 3b). After sequential high/medium/low-TF fine-tuning, prioritized fragments showed a clear bimodal distribution, with one peak near the canonical mononucleosome-protected length and a second peak enriched among shorter fragments (Fig. 3c). Given its strong performance across TF strata and biologically plausible fragment length enrichment (Fig. 3d; Fig. S8), this model was selected as the final Fragmentia-AI™ WGS model for all subsequent analyses.

### Evaluation of the model on the independent test cohort

**Fig. 4:**
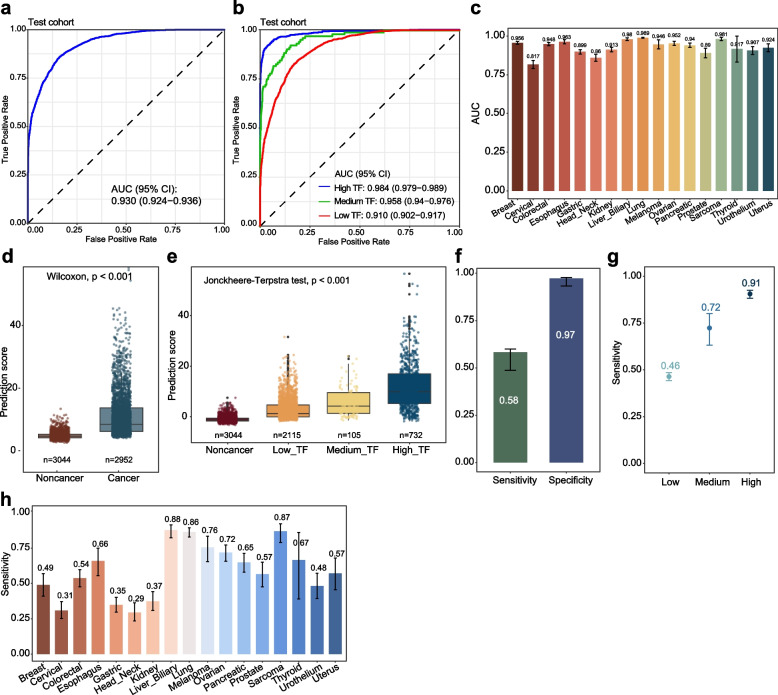
Evaluation of the model in the independent test cohort. **a** Receiver operating characteristic (ROC) curve for cancer detection in the independent test cohort. **b** ROC curves stratified by TF level (high, medium, and low). **c** Cancer type-specific areas under the curve (AUCs) in the test cohort. **d** Distribution of model prediction scores between noncancer and cancer samples. **e** Distribution of model prediction scores across noncancer samples and cancer samples stratified by TF level. **f** Overall sensitivity and specificity at the predefined operating threshold. **g** Sensitivity across TF strata at the predefined cutoff. **h** Cancer-type-specific sensitivity in the test cohort

We then assessed the generalizability of the final model in the independent test cohort. The model achieved an overall AUC of 0.930 (95% CI: 0.924–0.936) (Fig. 4a). AUCs were 0.984 (95% CI: 0.979–0.989) for high-TF, 0.958 (95% CI: 0.940–0.976) for medium-TF, and 0.910 (95% CI: 0.902–0.917) for low-TF samples (Fig. 4b). Cancer-type-specific analysis showed consistently high AUCs across tumor entities, with slightly reduced performance for cervical, gastric, head and neck, and prostate cancers (Fig. 4c). Prediction scores were significantly higher in cancer compared with noncancer samples (Wilcoxon test, *p* < 0.001) and increased monotonically with TF and clinical stage (Jonckheere–Terpstra test, *p* < 0.001) (Fig. 4d, e; Fig. S6e, f). Using the predefined threshold, the model achieved an overall sensitivity of 0.58 and specificity of 0.97 in the test cohort (Fig. 4f). Sensitivity increased with TF, from 0.46 in low-TF to 0.72 in medium-TF and 0.91 in high-TF samples, reflecting improved detectability with higher tumor fractions (Fig. 4g). Across cancer types, sensitivity varied substantially, with the highest observed for liver/biliary cancer (0.88), sarcoma (0.87), and lung (0.86), followed by melanoma (0.76) and ovarian (0.72) (Fig. 4h). Lower sensitivities were observed for kidney (0.37), gastric (0.35), cervical (0.31), and head and neck (0.29) cancers, indicating heterogeneous detectability across tumor entities.

### Generalizability test on the DELFI cohort

**Fig. 5:**
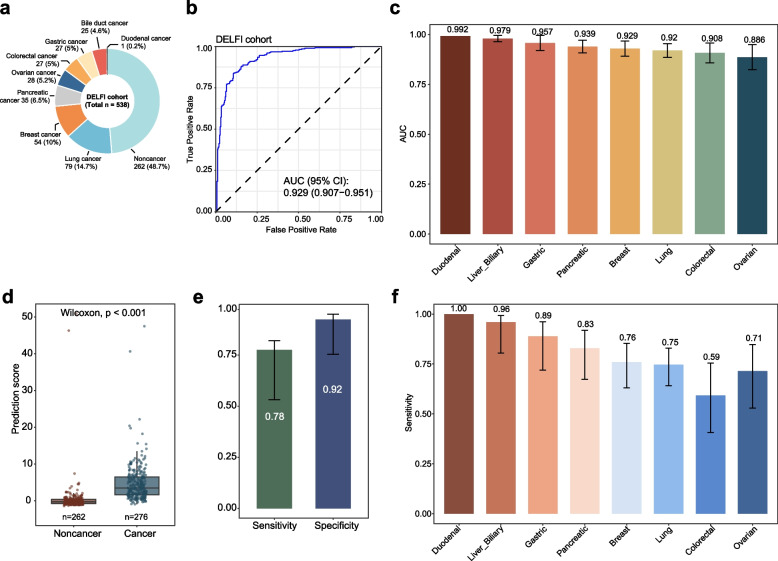
Model performance in the external public DELFI cohort. **a** Composition of the DELFI cohort, including healthy controls and cancer samples across multiple cancer types. **b** Receiver operating characteristic (ROC) curve for the model in the DELFI cohort. **c** Cancer type-specific areas under the curve (AUCs). **d** Distribution of model prediction scores between noncancer and cancer samples. **e** Overall sensitivity and specificity at the predefined threshold. **f** Cancer-type–specific sensitivity at the predefined threshold

To assess cross-platform generalizability, we evaluated the model in the public DELFI cohort, generated using a different sequencing platform and derived from a distinct population. The DELFI cohort included 538 subjects, comprising 262 noncancer controls (48.7%) and 276 cancer patients across eight tumor types (Fig. 5a). Despite substantial differences in sequencing technology and population characteristics, the model maintained strong discrimination performance, achieving an AUC of 0.929 (95% CI: 0.907–0.951), with consistently high performance across cancer entities (Fig. 5b, c). Prediction scores were significantly higher in cancer than in noncancer controls (Wilcoxon test, *p* < 0.001) (Fig. 5d). At the predefined cutoff threshold, the model achieved an overall sensitivity of 0.78 and specificity of 0.92, with sensitivities exceeding 0.59 across all cancer types (Fig. 5e, f). Collectively, these findings demonstrate that the learned cancer-associated fragmentomic representations generalize robustly across sequencing platforms and populations, supporting the broad transferability of the model.

### Model stability and repeatability

**Fig. 6:**
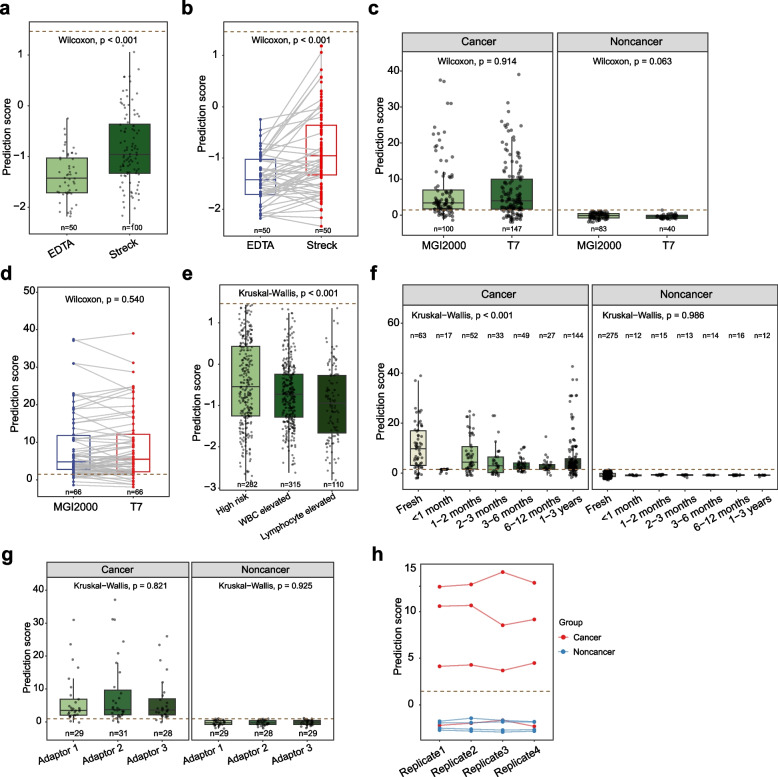
Analytical variability and technical reproducibility of the model. **a**, **b** Model prediction scores in noncancer samples stratified by plasma collection tube type are shown in independent samples (**a**) and paired samples from the same individuals (**b**). **c**, **d** Model prediction scores stratified by sequencing platform, shown in independent cancer and noncancer samples (**c**) and paired cancer samples from the same patients (**d**). **e–g** Model prediction scores stratified by disease context (e), plasma storage duration (**f**), and library adaptor type (**g**). **h** Technical repeatability was assessed using four aliquots from the same blood tube, processed by different operators across two sequencing batches. The dashed horizontal line indicates the predefined classification threshold corresponding to 98% specificity in the training cohort

Model robustness was evaluated using an analytical variability assessment cohort to assess whether common pre-analytical and experimental variables affected model predictions and classification performance. Across variations in blood collection tube type, sequencing platform, disease context, plasma freezing duration, library adaptor type, and technical repeats, the classification results remained stable at the predefined threshold (Fig. 6). All noncancer samples were consistently classified as negative, corresponding to a specificity of 100% across the evaluated conditions. Cancer samples also remained detectable, with sensitivities of 80.2% in the sequencing platform comparison, 80.8% in the plasma freezing duration analysis, and 90.9% in the library adaptor comparison.

Prediction scores were higher in Streck than in EDTA tubes among noncancer samples, both in the unpaired and paired analyses (Wilcoxon, *p* < 0.001; Fig. 6a, b). However, all scores remained below the predefined classification threshold, and tube-related variability can be controlled by using a consistent blood collection tube type within a given workflow. In cancer samples, paired analysis showed no significant difference between sequencing platforms (Wilcoxon, *p* = 0.540; Fig. 6d), supporting cross-platform consistency. Prediction scores also differed across noncancer disease contexts (Kruskal–Wallis, *p* < 0.001; Fig. 6e), but all samples were classified as negative. For plasma freezing duration, prediction scores varied across storage-duration groups in cancer samples (Kruskal–Wallis, *p* < 0.001; Fig. 6f), whereas no significant difference was observed in noncancer samples. This apparent association should be interpreted cautiously because the cancer storage-duration groups were not controlled for clinical variables that may influence ctDNA signal, including TF and disease stage. Prediction scores did not differ significantly across library adaptor types and were consistent across technical repeats (Fig. 6g, h). Overall, condition-associated score differences did not materially alter classification at the predefined threshold, supporting the analytical robustness of the model.

### Post-treatment model positivity reflects clinically relevant disease burden

Post-treatment model scores were evaluated for association with PFS in 143 patients with stage III/IV NSCLC receiving first-line immunotherapy plus platinum-based chemotherapy who achieved radiographic CR/PR by RECIST version 1.1 (Table S3).

**Fig. 7:**
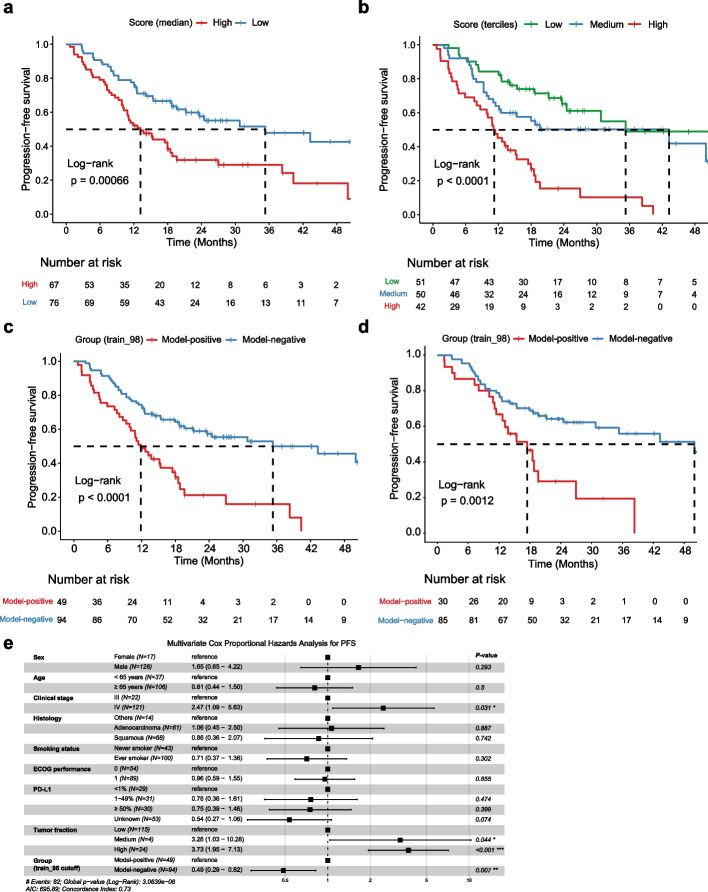
Association between model prediction score and PFS in advanced NSCLC. **a** Kaplan–Meier curves for progression-free survival (PFS) stratified by the median model prediction score in 143 patients with stage III/IV NSCLC who achieved radiographic complete or partial response after first-line immunotherapy combined with platinum-based chemotherapy. **b** Kaplan–Meier curves for PFS stratified by prediction score tertiles. **c** Kaplan–Meier curves for PFS stratified by the predefined cutoff corresponding to 98% specificity in the training cohort. Patients with prediction scores at or above this cutoff were classified as model-positive, whereas those with scores below the cutoff were classified as model-negative. **d** Kaplan–Meier curves for PFS in the low-TF (< 3%) subgroup, stratified by model status using the predefined cutoff. **e** Multivariable Cox proportional hazards model for PFS adjusted for sex, age, clinical stage, histology, smoking status, ECOG performance status, PD-L1 status, TF group, and model status. Hazard ratios are shown with 95% confidence intervals

When patients were stratified by the median prediction score, those in the high-score group exhibited significantly shorter PFS than those in the low-score group (log-rank *p* = 0.00066; Fig. 7a). Consistently, tertile-based stratification showed a graded separation of survival curves (log-rank *p* < 0.0001), with progressively shorter PFS from the low- to high-score groups (Fig. 7b). Using the predefined cutoff corresponding to 98% specificity in the training cohort, model-positive patients had significantly inferior PFS compared with model-negative patients (log-rank *p* < 0.0001; Fig. 7c), indicating that post-treatment model positivity captured clinically relevant residual tumor-derived fragmentomic signals rather than only a binary cancer detection readout.

Among patients with low TF (115/143, 80.4%; Table S3), model-positive low-TF patients continued to exhibit significantly shorter PFS than model-negative patients (log-rank *p* = 0.0012; Fig. 7d). In multivariate Cox analysis adjusting for sex, age, clinical stage, histology, smoking status, ECOG performance status, PD-L1 status, and TF group, model-negative status remained associated with improved PFS compared with model-positive status (HR = 0.49, 95% CI: 0.29–0.82; *p* = 0.007; Fig. 7e). Clinical stage and TF group were also associated with PFS in the multivariate model. Together, these findings indicate that post-treatment tumor-derived fragmentomic signals inform risk stratification among advanced NSCLC patients who achieved radiographic CR/PR after first-line chemoimmunotherapy.

## Discussion

In this study, we developed and validated Fragmentia-AI™ WGS, a mutation-calling-independent, reference-genome-based fragmentomic pipeline for pan-cancer detection using ULP-WGS of plasma-derived cfDNA. Unlike approaches requiring predefined mutation panels, high-depth somatic mutation calling, circulating protein biomarkers, or cancer-associated methylation profiling [5, 16–18], our model leverages genome-wide cfDNA fragmentation patterns directly from standard WGS without requiring additional molecular assays or specialized library preparation. It combines reference-genome-aligned fragments, stochastic sampling, Transformer encoding, attention-based MIL, and TF-guided sequential fine-tuning. This design enables cancer signal extraction from sparse data while maintaining predefined-threshold performance across TF strata, cancer types, and independent validation settings.

Sequential fine-tuning across TF strata addresses the reduced tumor-derived DNA abundance and increased dilution by hematopoietic cfDNA at low TF [19–23]. Models trained on high-TF or high/medium-TF samples performed well in high-signal settings but degraded as TF decreased. Progressive high/medium/low-TF fine-tuning improved low-TF discrimination (AUC: 0.884 in validation and 0.910 in the test cohort) without compromising high-TF performance. Fragment length analyses supported biological relevance: high-TF-only training produced a near-unimodal distribution, whereas sequential inclusion of medium- and low-TF samples produced a bimodal pattern with peaks near nucleosome-protected cfDNA (~ 167 bp) and shorter fragments (~ 100–150 bp) enriched for tumor-associated signals. This does not imply that all short fragments are tumor-derived; rather, the low-TF-adapted model preferentially captured fragment populations enriched for cancer-associated information. The pattern is consistent with tumor-specific fragmentation and the need to detect subtler signals when tumor-derived cfDNA is diluted by non-tumor cfDNA [1, 4, 24, 25]. This progressive shift toward shorter-fragment enrichment is also biologically plausible in the context of low-TF samples, where sparse tumor-derived cfDNA is diluted within abundant non-tumor cfDNA, requiring the model to rely on subtler fragmentation features that remain informative under weak-signal conditions.

Model generalizability was evaluated across multiple independent validation settings. Model performance was preserved in the independent multi-cancer test cohort and confirmed in the DELFI cohort, which was generated on a distinct platform and from a different population. Sequencing QC assessment showed negligible-to-small differences between cancer and noncancer samples and only weak correlations with model scores (Fig. S9), suggesting limited QC-associated influence on model performance. The analytical variability cohort further showed that common pre-analytical and experimental variables, including blood collection tubes, sequencing instruments, plasma freezing duration, library adaptors, and technical processing, did not materially alter classification at the predefined threshold. Across the evaluated conditions, noncancer samples were consistently classified as negative, while cancer sensitivity was maintained above 80% in the larger technical comparisons, supporting the model’s resilience to routine laboratory and pre-analytical variation.

Model performance nevertheless depended on tumor signal abundance. In the independent test cohort, sensitivity increased with TF, as higher-TF samples provide stronger fragmentomic signals, whereas low-TF signals are more diluted by non-tumor cfDNA and less likely to exceed a fixed high-specificity threshold. Thus, despite high low-TF AUC, many low-TF cancers—particularly early-stage cases—remained below the decision threshold. The model is therefore better positioned as a cost-efficient risk-stratification or rule-in tool than as a standalone test to exclude cancer in early-stage low-TF disease.

Similar considerations may also help explain the heterogeneous detectability observed across cancer types. Tumor-derived cfDNA release varies substantially among tumor entities and is influenced by tumor burden, vascularization, cell death, anatomical site, disease extent, and biological aggressiveness [1, 19]. Cancers with lower or more variable systemic ctDNA shedding may therefore generate weaker plasma-derived fragmentomic signals, especially under ultra-low-pass sequencing, where subtle deviations from the noncancer cfDNA background are more difficult to resolve. In addition, tissue-specific differences in chromatin organization, nucleosome positioning, and cfDNA fragmentation may affect the separability of tumor-derived fragmentomic profiles [3, 4, 25]. Therefore, the relatively lower performance observed for certain cancer types and in low-TF samples likely reflects overlapping biological and technical challenges associated with weak tumor-derived cfDNA signals. Future improvements should focus on strategies that enhance signal detection in weak-signal settings, including expanding the representation of low-TF and lower-detectability tumor types during model development, optimizing decision thresholds for specific clinical contexts, and integrating complementary biomarkers such as methylation, copy number alterations, or protein markers.

Beyond cancer detection, model prediction scores were significantly associated with PFS in patients with advanced NSCLC receiving first-line chemoimmunotherapy. Importantly, this relationship held whether using a data-driven cutoff or the predefined fixed threshold, with patients above the cutoff exhibiting shorter PFS independent of key clinical covariates. These findings suggest that the fragmentomic patterns captured by the model are associated with clinically meaningful differences in patient outcomes and may have potential utility for prognostic stratification and longitudinal disease monitoring. Although post-treatment model positivity may reflect clinically relevant residual tumor-associated fragmentomic signals, it may also be influenced by treatment-related cfDNA release, inflammation, tissue repair, immune-mediated response, hematopoietic changes, or other low TF-associated biological effects. Therefore, model positivity in this cohort should be interpreted as a prognostic cfDNA fragmentomic signal rather than direct evidence of molecular residual disease (MRD). The current model was not specifically developed or validated as a dedicated MRD assay, and its performance in postoperative or post-treatment settings with extremely low ctDNA levels remains to be established.

The novelty of Fragmentia-AI™ WGS lies in integrating reference-genome-based ULP-WGS fragmentomics, Transformer-based representation learning, TF-guided sequential fine-tuning, and fixed-threshold validation. ULP-WGS may lower sequencing costs, while cross-platform, cross-population, and condition-resilient performance supports use across institutions. Predefined thresholds improve clinical interpretability, and associations with outcomes suggest potential beyond binary detection. The learned representations may also support tissue-of-origin (TOO) prediction, although the current study was optimized for binary pan-cancer detection. Because TOO inference remains difficult in low-TF multi-cancer early detection settings [26, 27], future work could combine fragmentomics with methylation or copy-number features and multitask learning to jointly model cancer presence and tissue origin.

Several limitations should be acknowledged. First, the computational demands of Transformer-based models may restrict use in resource-constrained settings. Second, sensitivity remained limited in weak-signal contexts, including low-TF samples, early-stage low-TF cancers, and certain lower-detectability tumor types, making this a priority for future development. Third, TF-stratified performance could not be evaluated in the DELFI cohort because BAM files required for ichorCNA were unavailable. Fourth, although post-treatment model positivity was prognostic in advanced NSCLC, the model has not been optimized or validated for postoperative or post-treatment MRD settings with extremely low ctDNA and should be interpreted as a risk-stratification and monitoring tool rather than a definitive MRD assay. Finally, the analytical variability assessment was not a fully powered, prospectively balanced equivalence study. Prospective technical-control materials and balanced replicate designs are needed to quantify condition-specific variability.

In summary, this study demonstrates that cost-effective, ultra-sparse sequencing can achieve robust, generalizable cancer detection, with stable performance across platforms, populations, and technical conditions. The model’s ability to provide clinically interpretable prediction scores and to capture prognostic signals underscores its potential for scalable, minimally invasive liquid biopsy applications in precision oncology.

## Materials and methods

### Study cohorts and data acquisition

This study included seven independent cohorts: training, validation, independent test, public DELFI cross-platform validation [5], paired-sequencing low-TF evaluation, clinical outcome evaluation, and analytical variability assessment cohorts.

The training and validation cohorts were collected independently during non-overlapping periods from the same multi-center network: January 2021 to December 2023 for training and January 2024 to December 2025 for validation. These cohorts were sourced from Nanjing Drum Tower Hospital, First People’s Hospital of Changzhou, Taizhou Central Hospital (Taizhou University Hospital), Sun Yat-sen Memorial Hospital, Beijing Obstetrics and Gynecology Hospital, The First Affiliated Hospital of Nanjing Medical University, The First Affiliated Hospital of Army Medical University, The Fourth Affiliated Hospital of Nanjing Medical University, Cancer Hospital of the Chinese Academy of Medical Sciences, and Guangdong Provincial People’s Hospital. Together, they comprised 5,738 plasma samples, including 2,394 noncancer controls and 3,344 cancer patients across 17 cancer types. Only the first eligible baseline pretreatment sample per individual was retained; duplicate, repeated, longitudinal, and post-treatment samples were excluded.

The independent test cohort (*n* = 5,996) was collected from a distinct set of medical centers, including Eastern Theater Command Hospital, The First Affiliated Hospital of China Medical University, Zhongshan Hospital, Fudan University Shanghai Cancer Center, Second Xiangya Hospital of Central South University, Harbin Medical University Cancer Hospital, Fujian Medical University Union Hospital, and Affiliated Cancer Hospital of Xiangya School of Medicine. This cohort included 3,044 noncancer controls and 2,952 cancer patients and was independent of the training and validation cohorts at the sample, patient, medical-center, and sequencing-batch levels. No samples from the independent test cohort were used for model training, hyperparameter optimization, feature selection, threshold determination, or model selection. Cross-platform and population robustness were further evaluated using the public DELFI cohort, which comprised 262 noncancer controls and 276 cancer patients from the FinaleDB database (http://finaledb.research.cchmc.org/). An independent evaluation cohort of 143 patients with stage III/IV non-small cell lung cancer (NSCLC) was enrolled from Nanjing Drum Tower Hospital to examine the association between model prediction scores and progression-free survival (PFS). All patients received first-line immunotherapy combined with platinum-based chemotherapy and achieved radiographic complete response (CR) or partial response (PR) according to RECIST version 1.1. Plasma samples were collected at the time of radiographic response assessment.

An independent paired-sequencing cohort from the Geneseeq institutional repository was used to evaluate the biological relevance of WGS-defined low TF. It included 206 samples with ULP-WGS and matched GeneseeqPrime™ 425-gene panel data. All samples in this cohort had ichorCNA-estimated TF < 3% and were not used for model development, threshold determination, or selection.

The analytical variability assessment cohort comprised samples processed at Nanjing Geneseeq Technology Inc., with analysis subsets used to evaluate the effects of blood collection tube type (150 noncancer samples; 50 paired noncancer samples), sequencing platform (370 samples, including 247 cancer and 123 noncancer samples; 66 paired cancer samples), disease context (707 noncancer samples), plasma freezing duration (742 samples, including 385 cancer and 357 noncancer samples), and library adaptor type (174 samples, including 88 cancer and 86 noncancer samples). Technical reproducibility was assessed using four aliquots from each blood tube, processed by two operators across two sequencing batches.

### Blood processing, cfDNA extraction, and ULP-WGS library preparation

Preoperative, treatment-naive peripheral blood samples were collected in EDTA tubes (Becton Dickinson) and shipped on dry ice to a CLIA-certified and CAP-accredited centralized laboratory (Nanjing Geneseeq Technology Inc., Nanjing, China). Training, validation, independent test, and NSCLC cohorts used identical cfDNA extraction, library preparation, sequencing, and analysis protocols. The analytical variability cohort was processed under the specified alternative conditions.

In brief, plasma was isolated via a two-step centrifugation protocol, with an initial centrifugation at 1,600 × g for 10 min at 4℃, followed by a second centrifugation at 16,000 × g for 10 min at 4℃ to remove cellular debris. Cell-free DNA (cfDNA) was extracted using the QIAamp Circulating Nucleic Acid Kit (Qiagen Cat. No. 55114) and stored at −80℃ until further use. cfDNA concentration was quantified using the dsDNA HS assay kit on a Qubit 3.0 fluorometer (Life Technologies, US), and fragment size distribution was assessed using the Agilent Bioanalyzer 2100 system (Agilent Technologies, Santa Clara, USA). For each sample, 5–10 ng of cfDNA was used to construct a PCR-free whole-genome sequencing (WGS) library using the KAPA Hyper Prep kit (KAPA Biosystems). Institutional ULP-WGS libraries were primarily sequenced in paired-end mode with 150-bp reads on the DNBSEQ-T7 platform, with a small subset of samples in the analytical variability assessment cohort sequenced on MGI2000 for platform-variability evaluation. Training and validation cohorts were sequenced to approximately 0.1 × coverage, whereas the independent test, clinical evaluation, and analytical variability cohorts were sequenced to approximately 0.05 × coverage. The public DELFI cohort obtained from FinaleDB was originally generated using 100-bp paired-end whole-genome sequencing on the Illumina HiSeq 2000/2500 platform [5]. Tumor fraction (TF) was inferred through copy number analysis of plasma cfDNA using ichorCNA [15]. Given the reported limit of detection of approximately 3%, samples were classified as low TF (< 3%), medium TF (3–5%), or high TF (> 5%).

### Data preprocessing for genomic language modeling

Raw sequencing reads in BAM files were processed to extract high-quality cfDNA sequences. Paired-end reads with mapping quality scores of MAPQ ≥ 30 were retained, and fragments overlapping low-complexity regions, known repetitive elements, or standard genomic blacklist regions were excluded. Sequencing QC metrics used for technical-confounding assessment were calculated before model inference and were not used as model input features. To capture sequence motifs flanking the fragmentation sites, which are informative for cfDNA biology, we extracted the genomic coordinates of each fragment and extended them by 6 bp upstream of the start and 6 bp downstream of the end. This 6-bp extension was selected as a predefined short window to include the immediate sequence context surrounding cfDNA cleavage sites, while limiting the introduction of excessive neighboring genomic background. Prior studies have shown that breakpoint-adjacent motifs contain informative signals related to nuclease activity and chromatin organization [28, 29]. Including flanking bases also allows the transformer encoder to model sequence context across the cleavage boundary, rather than using only the internal fragment sequence. Using these adjusted coordinates, reference sequences were retrieved from the human reference genome GRCh37. Only fragments with a length ≤ 250 bp were retained to focus on mononucleosomal ctDNA characteristics.

### The Fragmentia-AI™ WGS framework

We developed a multiple instance learning (MIL) framework based on a DNABERT-2 foundation model [9] to detect cancer signals from ultra-sparse cfDNA data.

#### Stochastic Pool Sampling (SPS) data augmentation strategy

To address the high abundance of cfDNA fragments and improve training diversity, we implemented a Stochastic Pool Sampling (SPS) data augmentation strategy during model training. For each sample containing *N* fragments, a random subset of *K* unique instances (*K* < < *N*) was sampled at each epoch to construct a training “bag.” This process was repeated over *E* epochs, and the probability of sampling an identical fragment instance set across epochs is negligible.

$$\mathrm{P}(\mathrm{s}\mathrm{a}\mathrm{m}\mathrm{e} \;\mathrm{s}\mathrm{a}\mathrm{m}\mathrm{p}\mathrm{l}\mathrm{e}) \approx 1 - {e}^{\frac{-K(K-1)}{2M}}, \mathrm{w}\mathrm{h}\mathrm{e}\mathrm{r}\mathrm{e} \;\mathrm{M} = \frac{N!}{K! \times \left(N-K\right)!}$$

As a result, the model is exposed to a highly diverse representation of each patient’s cfDNA repertoire, while constraining the number of fragments processed per epoch, thereby improving computational efficiency and training stability.

#### Model architecture

The core architecture used a DNABERT-2 backbone followed by attention-based MIL pooling [30] and multilayer perceptron (MLP) classification. The encoder, pooling module, and MLP classifier were connected as a single model architecture, with parameter updates applied according to the stage-specific fine-tuning scheme described below.

- Tokenization & Encoding: Sampled DNA sequences were tokenized using Byte Pair Encoding (BPE) [31] and processed by DNABERT-2. The [CLS] token representation of the i-th sequence (z_i_ ∈ **ℝ**^**768**^) was extracted as the embedding.
- Attention Pooling: To aggregate instance-level embeddings (*Z* ∈ **ℝ**^***K***×**768**^, where *K* is the number of selected instances per sample) into a patient-level prediction, we employed a gated attention mechanism. Firstly, a fully connected layer (*W*_*1*_∈**ℝ**^**512×768**^) mapped *Z* to *H* via *H* = *ZW*_*1*_^⊤^ ∈ **ℝ**^***K×512***^ (all bias terms omitted for simplicity). The attention weight a_k_ for the k-th fragment was computed as:
  $${a}_{k}=\frac{\mathrm{e}\mathrm{x}\mathrm{p}\{\left(\mathrm{tanh}\left({H}_{k}{V}^{{\top}}\right) \odot \sigma \left({H}_{k}{U}^{{\top}}\right)\right){W}_{2}^{{\top}}\}}{\sum_{j=1}^{K}\mathrm{e}\mathrm{x}\mathrm{p}\{\left(\mathrm{tanh}\left({H}_{j}{V}^{{\top}}\right) \odot \sigma \left({H}_{j}{U}^{{\top}}\right)\right){W}_{2}^{{\top}}\}}$$

where *V* ∈ **ℝ**^**256×512**^, *U* ∈ **ℝ**^**256×512**^, *W*_*2*_ ∈ **ℝ**^**1×256**^**,** ⊙ denotes element-wise multiplication, and σ represents the sigmoid function.

The final patient representation *Y* ∈ **ℝ**^**1×512**^ is the weighted sum of instance embeddings:

$$\mathrm Y=\sum_{k=1}^K{a_kH}_k.$$

- Classification: The aggregate representation Y was passed through an MLP to generate a cancer probability score.

#### Progressive fine-tuning (curriculum learning)

The model was trained using a multi-stage progressive fine-tuning procedure rather than training all TF strata together in a single mixed-signal training procedure. Within each stage, the unfrozen DNABERT-2 parameters, attention-based MIL pooling module, and MLP classifier were optimized jointly by backpropagation, whereas the frozen DNABERT-2 parameters were kept fixed according to the stage-specific setting. To maximize sensitivity for early-stage cancer detection from cfDNA, we adopted a progressive fine-tuning (curriculum learning) strategy instead of conventional mixed-signal training. This approach leverages the higher signal-to-noise ratio of cancer samples with elevated tumor fraction (TF) to first learn fundamental sequence representations of tumor-derived cfDNA using a relatively small number of fragments. The model is then gradually adapted to increasingly challenging, low-signal scenarios by exposing it to samples with lower TF and larger fragment sets. This staged training prevents high-TF samples from dominating the loss function and improves the model’s ability to identify subtle cancer-associated signals in low-TF samples.

- Stage 1 (High-signal learning): The model was trained using cancer samples with high tumor fraction (TF > 5%) together with noncancer control samples to learn distinct cancer-associated sequence motifs and establish a robust decision boundary. The query, key, and value (QKV) weights of all 12 DNABERT-2 transformer layers, along with the downstream attention and MLP modules, were fine-tuned using *K* = 3,000 fragment instances per sample. Training was performed for up to 1,000 epochs.
- Stage 2 (Intermediate refinement): The model was further fine-tuned using samples with a medium tumor fraction (TF = 3–5%). The bottom six transformer layers were frozen, while the QKV weights of the top six transformer layers, together with the attention and MLP modules, were updated using *K* = 4,000 fragment instances per sample. Fine-tuning was conducted for up to 1,000 epochs.
- Stage 3 (Sensitivity boosting): Finally, the model was fine-tuned on low-tumor-fraction samples (TF < 3%) to enhance sensitivity in low-signal conditions. During this stage, the entire DNABERT-2 backbone was frozen, and only the attention and MLP modules were optimized using a larger input of K = 12,800 fragment instances per sample. Fine-tuning was carried out for up to 10,000 epochs.

### Model training and prediction

Model training employed a weighted binary cross-entropy loss and the AdamW optimizer. The learning rate was set to 1 × 10^–4^ for stages 1 and 2 and reduced to 5 × 10^–5^ for stage 3, with a ReduceLROnPlateau scheduler applied across all stages. A weight decay of 1 × 10^–3^ was used throughout training. To enable parameter-efficient fine-tuning, Low-Rank Adaptation (LoRA) [32] was applied to the query, key, and value (W_qkv_) matrices of the DNABERT-2 backbone, while the attention pooling and MLP layers were fully fine-tuned. All training was performed on NVIDIA B200 GPUs.

Prediction was performed using a fixed set of 128,000 fragment reads per sample. This input size was selected to balance predictive performance and computational efficiency, based on validation experiments showing that AUC improved as the number of inference reads increased from 16,000 to 128,000 and then largely plateaued, with minimal additional improvement at 256,000 or 512,000 reads (Fig. S10). To accommodate memory limits, prediction was executed in a two-stage, decoupled pipeline. First, during the encoding stage, fragments were processed in mini-batches by the DNABERT-2 encoder to generate fragment-level embeddings of dimension **ℝ**^**128,000×768**^. Second, during the aggregation stage, all embeddings were jointly processed by the attention pooling and MLP modules to produce a final cancer probability score. This decoupled prediction design enables scalable processing of large read counts and allows deployment on consumer-grade hardware without compromising performance.

### ctDNA enrichment analysis

To identify putative ctDNA fragments, we combined the attention scores from the attention pooling layer with the MLP-derived cancer probability scores to prioritize tumor-derived reads. Each cfDNA fragment was first encoded by the DNABERT-2 module into a fragment-level embedding (**ℝ**^**1×768**^), which was independently evaluated by the attention pooling and MLP modules. Fragments were initially ranked by their attention pooling scores, and the top 10% of fragments were retained as candidates with high tumor relevance. These candidate fragments were then further ranked by their MLP scores, and the top 10% within this candidate subset were selected. Thus, the final retained fragments represented approximately the top 1% of the original input fragments and were considered putative ctDNA-enriched fragments with the strongest model-inferred ctDNA-like signals.

### Statistical analysis

Statistical analyses were performed using R (v4.5.1) and Python (v3.11). Student’s t-tests or Wilcoxon rank-sum tests were used for comparing continuous variables, and Chi-square tests or Fisher’s exact tests were used for categorical variables, as appropriate. Receiver operating characteristic (ROC) analysis was conducted using the “pROC” R package, with the area under the curve (AUC) as the primary metric to evaluate model performance. The Jonckheere-Terpstra test was applied to evaluate monotonic trends across ordered groups. PFS was defined as the time from plasma sample collection at radiographic response assessment to progression or death, whichever occurred first. Patients without events were censored at the date of last follow-up. Kaplan–Meier curves were used to estimate the time-to-event distributions of subgroups, with statistical differences assessed using the log-rank test. Cox proportional hazards models were fitted to estimate hazard ratios (HR) with 95% confidence intervals (CI), and the proportionality of hazards was assessed using log(-log) survival plots. Potential technical confounding was assessed by comparing sequencing QC metrics between cancer and noncancer samples using Cohen’s d and by correlating QC metrics with model prediction scores using Spearman correlation. All *P*-values were two-sided, and *P* < 0.05 was considered statistically significant.

## Supplementary Information

Supplementary Material 1.

## Data Availability

The raw sequencing data generated and/or analyzed in this study are not publicly available due to participant privacy considerations, ethical requirements governing the use of human genomic data, and the substantial logistical and infrastructure requirements associated with storing, transferring, and managing large-scale whole-genome sequencing datasets. Qualified researchers may request access to the data and code through the following data use license request portal: https://zh.geneseeq.com/Fragmentia-AI-WGS/datause-license-request.html. Requests will be reviewed in accordance with applicable ethical, legal, and institutional requirements, and access may be granted after approval and completion of an appropriate data use agreement.
