# Supplementary material for: Generalizable cancer detection from ultra-low-pass WGS via deep contextual modeling of cfDNA sequences
Source: Mol Biomed. 2026 Aug 12;7:136. doi: 10.1186/s43556-026-00538-w (PMC13469060; doi:10.1186/s43556-026-00538-w)
Supplement: Supplementary file 1 [file 43556_2026_538_MOESM1_ESM.docx]

**Title: Generalizable Cancer Detection from Ultra-Low-Pass WGS via Deep Contextual Modeling of cfDNA Sequences**

Yang Xu^1#^, Song Wang^1#^, Guofeng Sun^1^, Yi Shen^1^, Shuang Chang^1^, Peng He^1^, Shuyu Wu^1^, Shanshan Yang^1^, Yuan Mao^2^, Zheng Wang^3^, Jiaqi Liu^4^, Tao Wang^5^, Bingzhong Zhang^6^, Kuirong Jiang^7^, Jierong Chen^8^, Hairong Huang^9^, Junjie Peng^10^, Xiaomeng Xia^11^, Yumei Wu^12^, Ming Wang^12^, Shun Xu^13^, Ji Tao^14^, Li Chong^15^, Junfei Zhu^16^, Daxin Huang^17^, Yuan Jiang^18^, Yongquan Wang^19^, Hua Bao^1*^, Xue Wu^1*^, Yang Shao^1, 20*^

1 Geneseeq Research Institute, Nanjing Geneseeq Technology Inc., Nanjing, China

2 The Fourth Affiliated Hospital of Nanjing Medical University, Nanjing, China

3 Department of Liver Surgery and Transplantation, Liver Cancer Institute, Zhongshan Hospital, Fudan University, Shanghai, China

4 State Key Laboratory of Molecular Oncology, National Cancer Center, Cancer Hospital of the Chinese Academy of Medical Sciences, Beijing, China

5 Department of Thoracic Surgery, Nanjing Drum Tower Hospital, Nanjing, China

6 Department of Gynecologic Oncology, Sun Yat-sen Memorial Hospital, Sun Yat-sen University, Guangzhou, China

7 Pancreas Center, The First Affiliated Hospital of Nanjing Medical University, Nanjing, China

8 Department of Clinical Laboratory, Guangdong Provincial People’s Hospital, Guangzhou, China

9 Department of Thoracic Surgery, Eastern Theater Command Hospital, Nanjing, China

10 Department of Colorectal Surgery, Fudan University Shanghai Cancer Center, Shanghai, China

11 Department of Gynaecology, Second Xiangya Hospital of Central South University, Changsha, China

12 Department of Gynecologic Oncology, Beijing Obstetrics and Gynecology Hospital, Beijing, China

13 Department of Thoracic Surgery, The First Affiliated Hospital of China Medical University, Shenyang, China

14 Department of Gastrointestinal Medical Oncology, Harbin Medical University Cancer Hospital, Harbin, China

15 Department of Respiratory Medicine, First People’s Hospital of Changzhou, Third Affiliated Hospital of Soochow University, Changzhou, China

16 Department of Respiratory and Critical Care Medicine, Taizhou Central Hospital (Taizhou University Hospital), Taizhou 318000, China

17 Department of Radiation Oncology, Fujian Medical University Union Hospital, Fuzhou, China

18 Clinical Laboratory of Hunan Cancer Hospital, Affiliated Cancer Hospital of Xiangya School of Medicine, Central South University, Changsha, China

19 Department of Urology, The First Affiliated Hospital of Army Medical University, Chongqing, China

20 School of Public Health, Nanjing Medical University, Nanjing, China

**# Yang Xu and Song Wang contributed equally to this work**

*** Co-corresponding authors**

**Yang Shao (**[**yang.shao@geneseeq.com**](mailto:yang.shao@geneseeq.com)**); Xue Wu (**[**xue.wu@geneseeq.com**](mailto:xue.wu@geneseeq.com)**); Hua Bao (**[**hua.bao@geneseeq.com**](mailto:hua.bao@geneseeq.com)**)**

**Supplementary Materials**

## **Supplementary Tables**

| **Table S1. Clinical characteristics of the training and validation cohorts** | | | | | | |
| --- | --- | --- | --- | --- | --- | --- |
|  | **Train (N = 3695)** | | **Validation (N = 2043)** | | **P-value** | |
|  | **Noncancer (N = 1245)** | **Cancer (N = 2450)** | **Noncancer (N = 1149)** | **Cancer (N = 894)** | **Noncancer** | **Cancer** |
| **Sex** | |  |  |  | 0.17 | 0.891 |
| Female | 638 (51.2%) | 1159 (47.3%) | 622 (54.1%) | 426 (47.7%) |  |  |
| Male | 607 (48.8%) | 1291 (52.7%) | 527 (45.9%) | 468 (52.3%) |  |  |
| **Age** |  |  |  |  |  |  |
| Median (range) | 56.0 [30.0, 77.0] | 60.0 [1.00, 92.0] | 56.0 [35.0, 76.0] | 60.0 [0, 84.0] | 0.0407 | 0.817 |
| < 60 years | 903 (72.5%) | 1211 (49.4%) | 844 (73.5%) | 425 (47.5%) |  |  |
| ≥ 60 years | 342 (27.5%) | 1239 (50.6%) | 305 (26.5%) | 469 (52.5%) |  |  |
| **Clinical stage** | |  |  |  | N/A | 0.935 |
| I | 0 (0%) | 717 (29.3%) | 0 (0%) | 263 (29.4%) |  |  |
| II | 0 (0%) | 770 (31.4%) | 0 (0%) | 272 (30.4%) |  |  |
| III | 0 (0%) | 194 (7.9%) | 0 (0%) | 75 (8.4%) |  |  |
| IV | 0 (0%) | 769 (31.4%) | 0 (0%) | 284 (31.8%) |  |  |
| **Cancer type** | |  |  |  | N/A | 0.0752 |
| Breast | 0 (0%) | 122 (5.0%) | 0 (0%) | 33 (3.7%) |  |  |
| Cervical | 0 (0%) | 194 (7.9%) | 0 (0%) | 65 (7.3%) |  |  |
| Colorectal | 0 (0%) | 192 (7.8%) | 0 (0%) | 90 (10.1%) |  |  |
| Esophagus | 0 (0%) | 76 (3.1%) | 0 (0%) | 31 (3.5%) |  |  |
| Gastric | 0 (0%) | 248 (10.1%) | 0 (0%) | 86 (9.6%) |  |  |
| Head_Neck | 0 (0%) | 156 (6.4%) | 0 (0%) | 58 (6.5%) |  |  |
| Kidney | 0 (0%) | 168 (6.9%) | 0 (0%) | 56 (6.3%) |  |  |
| Liver_Biliary | 0 (0%) | 152 (6.2%) | 0 (0%) | 69 (7.7%) |  |  |
| Lung | 0 (0%) | 322 (13.1%) | 0 (0%) | 111 (12.4%) |  |  |
| Melanoma | 0 (0%) | 87 (3.6%) | 0 (0%) | 26 (2.9%) |  |  |
| Ovarian | 0 (0%) | 158 (6.4%) | 0 (0%) | 79 (8.8%) |  |  |
| Pancreatic | 0 (0%) | 158 (6.4%) | 0 (0%) | 64 (7.2%) |  |  |
| Prostate | 0 (0%) | 116 (4.7%) | 0 (0%) | 40 (4.5%) |  |  |
| Sarcoma | 0 (0%) | 89 (3.6%) | 0 (0%) | 24 (2.7%) |  |  |
| Thyroid | 0 (0%) | 25 (1.0%) | 0 (0%) | 12 (1.3%) |  |  |
| Urothelium | 0 (0%) | 105 (4.3%) | 0 (0%) | 34 (3.8%) |  |  |
| Uterus | 0 (0%) | 82 (3.3%) | 0 (0%) | 16 (1.8%) |  |  |

| **Table S2. Clinical characteristics of the test cohort (N = 5996)** | | |
| --- | --- | --- |
|  | **Noncancer (N=3044)** | **Cancer (N=2952)** |
| **Sex** | |  |
| Female | 1626 (53.4%) | 1427 (48.3%) |
| Male | 1418 (46.6%) | 1525 (51.7%) |
| **Age** |  |  |
| Median (range) | 56.0 [25.0, 83.0] | 60.0 [3.00, 91.0] |
| < 60 years | 2258 (74.2%) | 1455 (49.3%) |
| ≥ 60 years | 786 (25.8%) | 1497 (50.7%) |
| **Clinical stage** | |  |
| I | 0 (0%) | 898 (30.4%) |
| II | 0 (0%) | 982 (33.3%) |
| III | 0 (0%) | 234 (7.9%) |
| IV | 0 (0%) | 838 (28.4%) |
| **Cancer type** | |  |
| Breast | 0 (0%) | 145 (4.9%) |
| Cervical | 0 (0%) | 224 (7.6%) |
| Colorectal | 0 (0%) | 253 (8.6%) |
| Esophagus | 0 (0%) | 88 (3.0%) |
| Gastric | 0 (0%) | 311 (10.5%) |
| Head_Neck | 0 (0%) | 190 (6.4%) |
| Kidney | 0 (0%) | 198 (6.7%) |
| Liver_Biliary | 0 (0%) | 194 (6.6%) |
| Lung | 0 (0%) | 420 (14.2%) |
| Melanoma | 0 (0%) | 86 (2.9%) |
| Ovarian | 0 (0%) | 231 (7.8%) |
| Pancreatic | 0 (0%) | 194 (6.6%) |
| Prostate | 0 (0%) | 122 (4.1%) |
| Sarcoma | 0 (0%) | 100 (3.4%) |
| Thyroid | 0 (0%) | 12 (0.4%) |
| Urothelium | 0 (0%) | 114 (3.9%) |
| Uterus | 0 (0%) | 70 (2.4%) |

| **Table S3. Baseline characteristics of NSCLC patients receiving chemoimmunotherapy (N = 143)** | |
| --- | --- |
| **Characteristic** | N (%) |
| **Sex** |  |
| Female | 17 (11.9%) |
| Male | 126 (88.1%) |
| **Age** |  |
| Median (range) | 66.0 [47.0, 78.0] |
| < 65 years | 37 (25.9%) |
| ≥ 65 years | 106 (74.1%) |
| **Clinical stage** |  |
| III | 22 (15.4%) |
| IV | 121 (84.6%) |
| **Histology** |  |
| Squamous | 68 (47.5%) |
| Adenocarcinoma | 61 (42.7%) |
| Others | 14 (9.8%) |
| **Smoking status** |  |
| Ever smoker | 100 (69.9%) |
| Never smoker | 43 (30.1%) |
| **ECOG performance status** |  |
| 0 | 54 (37.8%) |
| 1 | 89 (62.2%) |
| **PD-L1 status** |  |
| < 1% | 29 (20.3%) |
| 1-49% | 31 (21.7%) |
| ≥ 50% | 30 (21.0%) |
| Unknown | 53 (37.0%) |
| **Tumor fraction** |  |
| Median (IQR) | 1.03% (0.71% - 1.94%) |
| Low-TF (< 3%) | 115 (80.4%) |
| Medium-TF (3-5%) | 4 (2.8%) |
| High-TF (≥ 5%) | 24 (16.8%) |
| **Radiographic response** |  |
| CR | 5 (3.5%) |
| PR | 138 (96.5%) |
| Abbreviations: ECOG, Eastern Cooperative Oncology Group; PD-L1, Programmed Cell Death Ligand 1; IQR, interquartile range; TF, tumor fraction; CR, complete response; PR, partial response | |

| **Table S4. Sensitivity of low-TF samples stratified by clinical stage group and cohort** | | | | |
| --- | --- | --- | --- | --- |
| **Dataset** | **Stage group** | **No. of low-TF cancer samples** | **No. of cancer samples detected as positive** | **Sensitivity (95% CI)** |
| Train | I/II | 1254 | 496 | 0.396 (0.368 – 0.423) |
| Train | III/IV | 366 | 262 | 0.716 (0.667 – 0.762) |
| Validation | I/II | 431 | 171 | 0.397 (0.350 – 0.445) |
| Validation | III/IV | 126 | 86 | 0.683 (0.594 – 0.763) |
| Test | I/II | 1628 | 637 | 0.391 (0.367 – 0.415) |
| Test | III/IV | 487 | 345 | 0.708 (0.666 – 0.748) |
| Abbreviation: TF, tumor fraction; CI, confidence interval | | | | |

## **Supplementary Figures**

### **Figure S1. Distribution of tumor fraction groups and clinical stages across cohorts**

(**a-c**) Pie charts showing the proportions of samples classified as High_TF, Medium_TF, and Low_TF in the training (a), validation (b), and test (c) cohorts. (**d-f**) Clinical stage distributions within each tumor fraction group in the training (d), validation (e), and test (f) cohorts. Significance annotations indicate chi-square test results after Benjamini-Hochberg correction for multiple comparisons.

### **Figure S2. Cancer type distribution across tumor fraction groups by cohorts**

Heatmap showing the distribution of cancer types across high-, medium-, and low-tumor-fraction (TF) groups in the training, validation, and test cohorts. The colour scale represents the proportion of each cancer type within each TF group, and each tile is labelled with the corresponding sample count and percentage.

### **Figure S3. Orthogonal validation of WGS-defined low-TF samples using matched targeted panel sequencing**

(**a**) Distribution of panel-derived variant allele frequency (VAF) in an independent cohort with paired ULP-WGS and matched targeted panel sequencing data (GeneseeqPrime™, 425-gene panel; n = 206). All samples in this cohort had ichorCNA-estimated TF <3% based on ULP-WGS. (**b**) Fragmentia-AI WGS sensitivity across panel-derived VAF strata.

### **Figure S4. Model performance after fine-tuning on high-tumor fraction samples**

(**a, b**) Receiver operating characteristics (ROC) curves of the model fine-tuned on high-TF samples in the training (a) and validation (b) cohorts. (**c**) ROC curves for the validation cohort stratified by tumor fraction. (**d, e**) Box plots showing the distribution of model prediction scores between noncancer and cancer patients across cohorts. (**f**) Box plots showing the distribution of model prediction scores between noncancer and cancer patients stratified by tumor fraction. (**g**) Distribution of prediction scores across ordered tumor fraction strata in the validation cohort.

### **Figure S5. Model performance after fine-tuning on high- and medium-tumor fraction samples**

(**a, b**) Receiver operating characteristics (ROC) curves of the model fine-tuned on high- and medium-TF samples in the training (a) and validation (b) cohorts. (**c, d**) Box plots showing the distribution of model prediction scores between noncancer and cancer patients stratified by tumor fraction in the training (c) and validation (d) cohorts. (**e**) Distribution of prediction scores across ordered tumor fraction strata in the validation cohort.

### **Figure S6. Prediction score distributions of the final model stratified by tumor fraction and clinical stage across cohorts**

Box plots showing the distribution of Fragmentia-AI WGS model prediction scores in samples ordered by tumor fraction or clinical stage across cohorts.

### **Figure S7. Cancer-type-specific sensitivity in low-TF cancer samples across cohorts**

The proportion of low-tumor-fraction (TF) cancer samples classified as positive at the predefined threshold is shown for each cancer type in the training, validation and test cohorts. Each point represents one cancer type within a cohort and is positioned according to its positive detection rate. Labels indicate the number of positive samples over the total number of low-TF cancer samples, with the corresponding percentage shown in parentheses. Point colour denotes the low-TF sample size category.

### **Figure S8. Fragment length distribution of the Fragmentia-AI WGS model across cancer types in the validation cohort**

Density plots comparing the top 1% cfDNA fragments (blue) selected by the Fragmentia-AI WGS model with 1% randomly selected fragments (yellow) in the validation cohort, stratified by cancer type.

### **Figure S9. Assessment of sequencing quality metrics as potential technical confounders**

(**a-c**) Distributions of sequencing QC metrics, including adapter_dimer_pct, clean_reads, coverage coefficient of variation (CV), q20_percentage, q30_percentage, and ratio_of_mapped, between noncancer and cancer samples in the training (a), validation (b), and test (c) cohorts. Cohen’s d was used to quantify standardized group differences. (**d**) Spearman correlations between each sequencing QC metric and model prediction scores across the training, validation, and test cohorts. Asterisks indicate statistical significance (*p < 0.05, **p < 0.01, ***p < 0.001).

### **Figure S10. ROC curves for model performance with different numbers of inference reads**

Receiver operating characteristic (ROC) curves are shown for validation samples (n = 2,043) using 16,000, 32,000, 64,000, 128,000, 256,000, and 512,000 fragment reads.
